# Cobalt Alleviates GA-Induced Programmed Cell Death in Wheat Aleurone Layers via the Regulation of H_2_O_2_ Production and Heme Oxygenase-1 Expression

**DOI:** 10.3390/ijms151121155

**Published:** 2014-11-14

**Authors:** Mingzhu Wu, Jiale Li, Fangquan Wang, Feng Li, Jun Yang, Wenbiao Shen

**Affiliations:** 1 College of Life Sciences, Laboratory Center of Life Sciences, Nanjing Agricultural University, Nanjing 210095, China; E-Mails: mingzhuwus@126.com (M.W.); 2012816150@njau.edu.cn (J.L.); qiezi324@163.com (F.W.);; 2 China Tobacco Gene Research Center, Zhengzhou Tobacco Research Institute of China National Tobacco Corporation, Zhengzhou 450001, China; E-Mails: likite2002@163.com (F.L.); yangjun@ztri.com.cn (J.Y.)

**Keywords:** aleurone layers, cobalt, heme oxygenase-1, H_2_O_2_, programmed cell death, *Triticum aestivum*

## Abstract

Heme oxygenase-1 (HO-1) and hydrogen peroxide (H_2_O_2_) are key signaling molecules that are produced in response to various environmental stimuli. Here, we demonstrate that cobalt is able to delay gibberellic acid (GA)-induced programmed cell death (PCD) in wheat aleurone layers. A similar response was observed when samples were pretreated with carbon monoxide (CO) or bilirubin (BR), two end-products of HO catalysis. We further observed that increased HO-1 expression played a role in the cobalt-induced alleviation of PCD. The application of HO-1-specific inhibitor, zinc protoporphyrin-IX (ZnPPIX), substantially prevented the increases of HO-1 activity and the alleviation of PCD triggered by cobalt. The stimulation of HO-1 expression, and alleviation of PCD might be caused by the initial H_2_O_2_ production induced by cobalt. qRT-PCR and enzymatic assays revealed that cobalt-induced gene expression and the corresponding activities of superoxide dismutase (SOD), catalase (CAT) and ascorbate peroxidase (APX), three enzymes that metabolize reactive oxygen species, were consistent with the H_2_O_2_ accumulation during GA treatment. These cobalt responses were differentially blocked by co-treatment with ZnPPIX. We therefore suggest that HO-1 functions in the cobalt-triggered alleviation of PCD in wheat aleurone layers, which is also dependent on the enhancement of the activities of antioxidant enzymes.

## 1. Introduction

In cereals, the aleurone layer is a secretory tissue that plays an important role in seed germination. Gibberellic acid (GA) produced by the growing embryo during seed germination brings about many of the changes, for example the synthesis of hydrolytic enzymes, such as α-amylase, which occurs in cereal aleurone cells during endosperm mobilization [1,2,3]. Such hydrolytic enzymes degrade the storage reserves in the starchy endosperm and provide nutrients for the growing embryo. The aleurone layer is programmed to die following germination [4,5]. It was well-known that GA initiates programmed cell death (PCD) and that abscisic acid (ABA) can prevent PCD [4]. Detailed investigations have demonstrated that GA-induced PCD in barley aleurone layers is preceded by a decrease in the levels of antioxidant enzymes and an increase in the concentrations of endogenous reactive oxygen species, particularly hydrogen peroxide (H_2_O_2_) [5,6]. Thus, it has been deduced that H_2_O_2_ is a key mediator in GA-induced PCD in aleurone layers.

Cobalt, a component of vitamin B_12_, is an essential cofactor of several key enzymes and co-enzymes. Studies have shown that the B_12_ coenzyme content of *R. meliloti* cells is positively correlated with the cobalt content of the culture medium [7]. The requirement of cobalt in *R. meliloti* is greater when grown with nitrate, as compared to when ammonium is the only source of nitrogen in the culture medium [8]. Cobalt has also been shown to affect the growth and metabolism of plants to differing degrees, depending on the concentration and status of cobalt in the rhizosphere. For example, high concentrations of cobalt were able to inhibit starch grain differentiation and alter the structure and number of chloroplasts per unit area of leaf [9]. Previous studies also demonstrated that low doses of cobalt, applied in the form of fertilizer or as a presoaking or pre-sowing drench, were able to increase the yields of various crops [10,11]. The induction of adventitious rooting and lateral root formation triggered by cobalt was reported in cuttings of tomato and cucumber [12], and in seedlings of rice and tomato [13,14]. Low doses of cobalt can also retard senescence in lettuce leaves in darkness [15], increase drought resistance in seeds of *Aesculus hippocastanum* [16], and regulate alkaloid accumulation in medicinal plants [17]. However, the molecular mechanisms and the regulation of metabolic pathways underlying plant responses to cobalt have not been characterized in detail.

H_2_O_2_ and heme oxygenase (HO-1), an enzyme that catalyzes the degradation of heme groups to produce carbon monoxide (CO), biliverdin IX (BV) and free iron (Fe^2+^), have each been individually identified as fundamental signaling molecules or components that control a diverse range of physiological processes in animals and plants [18,19]. For example, H_2_O_2_ acts as a key regulator of multiple physiological processes in plants, including growth, differentiation, senescence and stress responses [20]. HO-1 functions in anti-inflammatory, anti-apoptotic and anti-proliferative roles in animal cells, and accumulating evidence suggests the importance of HO-1 in plant cells, as well [21,22,23,24]. BV, an initial degradation product of HO, is rapidly reduced by cytosolic biliverdin reductase to form the potent antioxidant, bilirubin (BR) [25]. CO has profound effects on intracellular signaling processes, such as the maintenance of ROS homeostasis in different plants that have been subjected to oxidative stress [26,27,28,29]. In plants, H_2_O_2_ production and *HO-1* gene expression are known to be induced by some phytohormones and environmental stimuli [23,30,31,32,33,34]. When investigated using a pharmacological approach, the synergistic effects of HO-1 and H_2_O_2_ were observed in plant acclimation responses to oxidative stress [35,36]. However, little is currently known about the interrelationship between HO-1 and H_2_O_2_ signaling in wheat aleurone layers.

Here, we examined the rapid induction of *HO-1* gene expression and H_2_O_2_ production following the treatment of wheat aleurone layers with cobalt. Further analysis showed that the cobalt-induced alleviation of GA-induced PCD is associated with the up-regulation of HO-1 expression. Thus, our study reveals a deeper layer of understanding of the molecular mechanism(s) through which cobalt functions in the regulation of plant developmental processes.

## 2. Results

### 2.1. Cobalt Delays Gibberellic Acid (GA)-Induced Programmed Cell Death (PCD)

**Figure 1:**
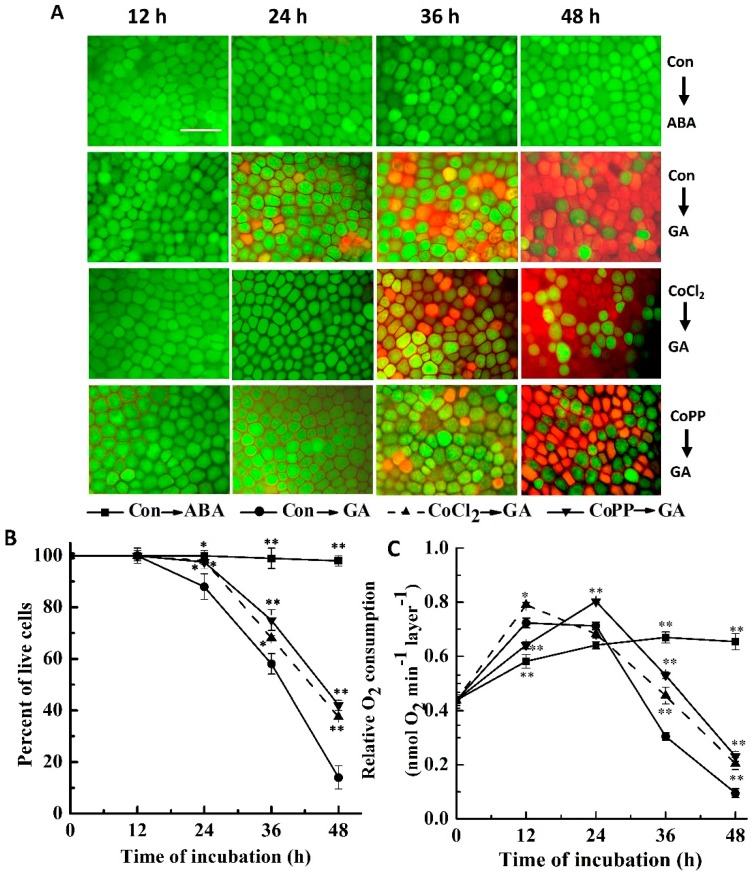
Gibberellic acid (GA)-induced programmed cell death is delayed by cobalt. Wheat aleurone layers were pretreated with distilled water (Con), 50 μM cobalt chloride (CoCl_2_) or 50 μM cobalt protoporphyrin IX (CoPP) for 6 h and then transferred to a medium containing 5 mM CaCl_2_ and 50 μM GA or abscisic acid (ABA). Digital images of fluorescently-labeled wheat aleurone cells are shown in (**A**); Live cells appear green and dead cells appear orange or red; Scale bar = 200 μm. The viability of cells following different treatments was quantified from at least four aleurone layer samples (**B**); Ten aleurone layers were placed in 3 mL of sterile water for the measurement of O_2_ consumption at the indicated time points (**C**). Data are the means ± Standard deviation (SD) of at least four independent measurements from different experiments. Bars with asterisks are significantly different as compared with the corresponding GA alone-treated samples, according to Student’s *t*-tests (* *p* < 0.05 or ** *p* < 0.01).

In accordance with previously reported results on barley and wheat aleurone layers [5,37,38], we evaluated the changes of cell viability using fluorescein diacetate (FDA) and *N*-(3-triethylammoniumpropyl)-4-(6-[4-(diethylamino) phenyl]-hexatrienyl) pyridinium dibromide (FM 4-64) staining. Normally, FDA is used to detect living cells (green fluorescence) [37]. Freshly prepared aleurone cells contain hundreds of small protein storage vacuoles. Following GA treatment, these vacuoles coalesced until one large protein storage vacuole nearly filled the cell [4]. The fluorescent probe, FM 4-64, can stain vacuolar membranes; it partitions slowly in live aleurone cells, but accumulates rapidly into dead cells and shows orange-red fluorescence (Figure 1A) [5,39,40,41]. Consistent with results from previous studies [4,5,38], GA-induced PCD observed in wheat aleurone layers in our experiments became more distinct with time (48 h period; Figure 1A). For example, the percentage of live cells was reduced to 14.0% ± 4.0% after 48 h of incubation in the treatment with GA solution, in comparison with 98.0% ± 2.0% live cells in ABA-treated samples (Figure 1B). Meanwhile, 92.0% ± 1.0% of the cells remained alive when wheat aleurone layers were incubated in the control CaCl_2_ solution (Figure S1).

We pretreated aleurone layers with various concentrations of cobalt solutions prepared with either cobalt chloride (CoCl_2_) or cobalt protoporphyrin IX (CoPP), followed by GA treatment, and then monitored cell viability. We used two different cobalt compounds to ensure that it was cobalt, rather than chloride ions or protoporphyrin IX, that affected the viability of wheat aleurone cells. As shown in Figure 1 and Figure S2, GA-induced PCD was significantly alleviated by pretreatment with 50 μM CoCl_2_ or 50 μM CoPP, in a time-dependent fashion. By contrast, no apparent alleviation of cell death was observed when 5 μM CoCl_2_ or 5 μM CoPP were used as pretreatments. At a higher concentration of CoCl_2_ or CoPP (500 μM), the percentage of dead cells in the aleurone layer was much higher, with at least 97% cell death, as compared with approximately 90% lethality in the GA-alone samples. However, no significant changes or weaker decreases in live cells were observed in the above three concentrations of CoCl_2_ or CoPP alone-treated for 48 h, compared with ABA alone (Figure S3). These results demonstrated that 50 μM cobalt delayed GA-induced PCD (Figure 1A,B and Figure S2). The response of relative O_2_ consumption displayed similar tendencies (Figure 1C). Therefore, 50 μM concentrations of CoCl_2_ and CoPP were used as treatments in the subsequent experiments.

### 2.2. Up-Regulation of α-Amylase Caused by Cobalt

**Figure 2:**
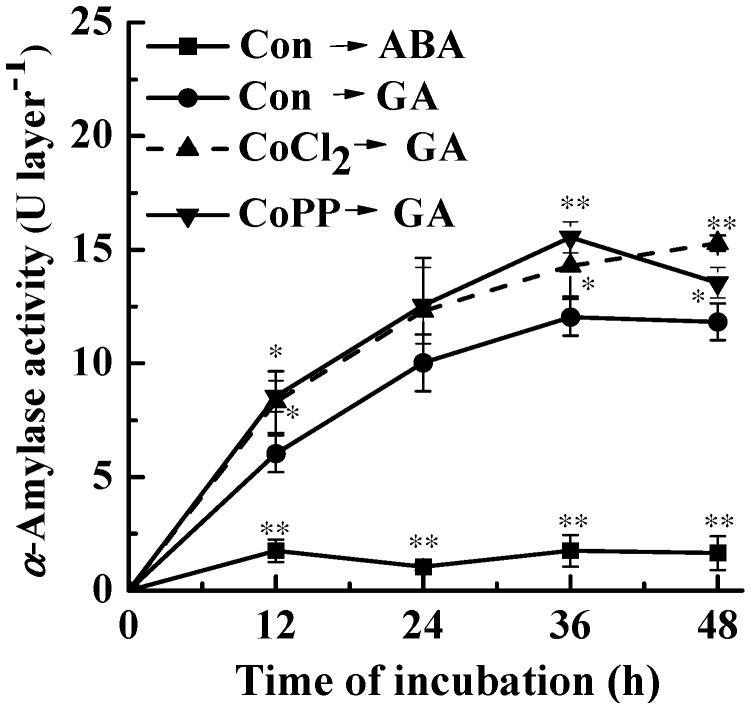
Cobalt affects GA-induced α-amylase activity. Wheat aleurone layers were pretreated with distilled water (Con), 50 μM CoCl_2_ or 50 μM CoPP for 6 h and then transferred to a medium containing 5 mM CaCl_2_ and 50 μM GA or ABA. α-Amylase activity was determined at the indicated times following various treatments. Data are the means ± SD of at least four independent measurements from different experiments. Within the same treatment time points, bars with asterisks are significantly different as compared with corresponding GA alone-treated samples, according to Student’s *t*-tests (* *p* < 0.05 and ** *p* < 0.01).

To evaluate if cobalt’s effect in delaying PCD results from a global inhibition or induction of GA-induced responses, we investigated the activity of α-amylase secreted into the incubation medium during 48 h of incubation of wheat aleurone layers with various treatments. As shown in Figure 2, we found that GA stimulated and ABA inhibited the secretion of α-amylase and that the accumulation of α-amylase activity in the incubation medium ceased 36 h after the incubation in GA-alone treated samples. In comparison with GA-alone samples, significant increases of α-amylase activity were observed in CoCl_2_- and CoPP-pretreated samples during a similar treatment time. These above results suggested that CoCl_2_- and CoPP-induced delay of PCD was not a result of a global inhibition of GA-induced responses.

### 2.3. Stimulation of HO-1 Gene Expression and H_2_O_2_ Production by Cobalt

**Figure 3:**
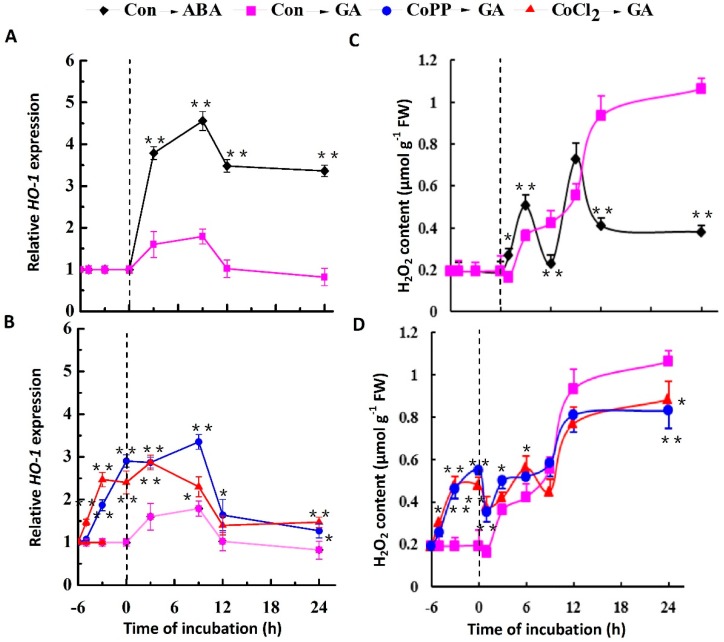
Induction of *HO-1* gene expression and H_2_O_2_ production in response to cobalt. Wheat aleurone layers were pretreated with distilled water (Con), 50 μM CoCl_2_ or 50 μM CoPP for 6 h (marked as “6” on the graph), and then transferred to a medium containing 5 mM CaCl_2_ and 50 μM GA or ABA. Wheat *HO-1* transcripts (**A**,**B**) and H_2_O_2_ content (**C**,**D**) were measured at the indicated time points. Data are the means ± SD of three independent measurements from different experiments. Within the same treatment time points, bars with asterisks are significantly different as compared with corresponding GA-alone samples, according to Student’s *t*-tests (* *p* < 0.05 and ** *p* < 0.01).

Cobalt is known to be a transcriptional activator of HO-1 [42]. To further understand the molecular mechanisms of cobalt’s role in the physiology of wheat aleurone, we undertook a detailed study of cobalt-induced *HO-1* gene expression. We also evaluated the effect of ABA and GA on *HO-1* gene expression. As shown in Figure 3A, HO-1 transcription increased sharply in ABA-treated wheat aleurone layers, with a significant burst of expression seen at the 9 h time point, followed by a gradual decrease by the 12 h time point. Subsequently, the ABA-treated samples maintained a higher level of HO-1 transcription as compared with GA-treated samples. The treatment with GA alone brought about a similar, though weaker, induction during the initial 12 h of incubation (Figure 3A,B). Stimulation of *HO-1* gene expression by cobalt pretreatment was observed (Figure 3B).

In animals, several lines of evidence point to the fact that H_2_O_2_ and HO-1 not only modulate each other’s functions within the cells, but also depend on each other to impart their effects in most conditions [43]. We therefore measured H_2_O_2_ content in wheat aleurone layers. A biphasic burst of H_2_O_2_ was observed in the ABA-treated samples (Figure 3C). Peaks of H_2_O_2_ production were observed at 3 and 9 h after ABA treatment. In contrast, in the GA-treated samples, the H_2_O_2_ concentration increased continuously throughout the 24 h treatment period. In the samples pretreated with cobalt, the higher level of H_2_O_2_ production induced by cobalt pretreatment was sustained during the initial 9 h following the addition of GA (Figure 3D). Subsequently, in comparison with the GA alone-treated samples, H_2_O_2_ production was decreased in the cobalt pretreated samples. The inhibition at 24 h was partial and never exceeded four-fifths of that observed in the GA-alone samples.

### 2.4. Exogenous H_2_O_2_ Mimics the Effect of Cobalt on Delaying GA-Induced PCD

**Figure 4:**
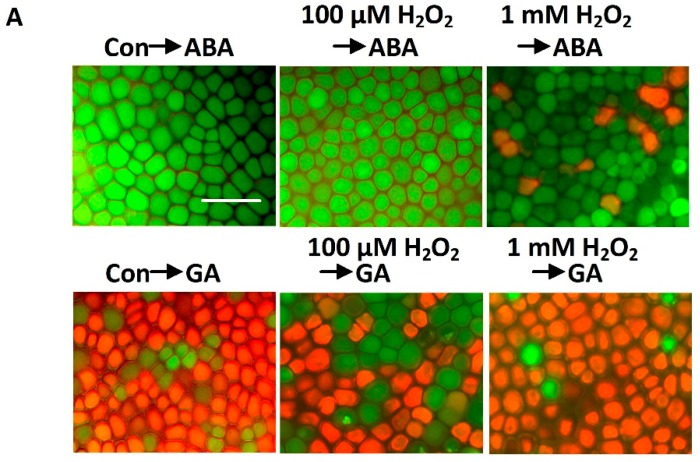

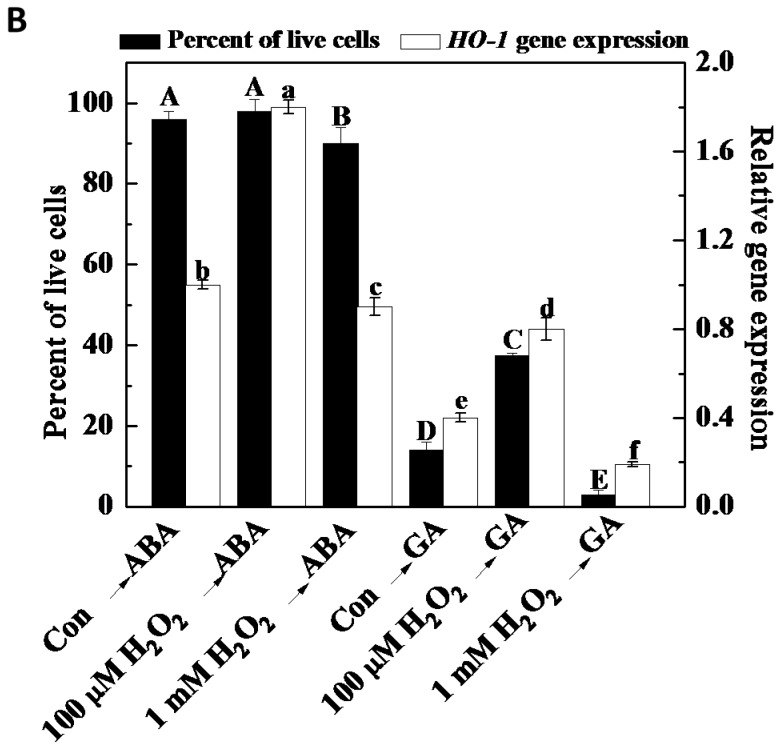
The effect of H_2_O_2_ pretreatment on GA-induced PCD. Wheat aleurone layers were pretreated with distilled water (Con), 100 μM H_2_O_2_ or 1 mM H_2_O_2_ for 6 h and then transferred to a medium containing 5 mM CaCl_2_ and 50 μM GA or ABA. Digital images of fluorescently-labeled wheat aleurone cells after 48 h of incubation are shown in (**A**), live cells appear green and dead cells appear orange or red; Scale bar = 200 μm; The viability of cells following different treatments was quantified after 48 h of incubation from at least four aleurone layers (**B**, **left**). Wheat *HO-1* transcripts were measured after 12 h of treatment (**B**, **right**). Data are the means ± SD of at least four independent measurements from different experiments. Bars denoted by the different letters are significantly different at *p* < 0.05, according to Duncan’s multiple test.

H_2_O_2_ is known to act as a key regulator of multiple physiological processes in plants, including growth, differentiation, senescence and stress responses [20]. In our experiments, an initial H_2_O_2_ up-regulation was observed in cobalt-pretreated samples (Figure 3D). In order to further evaluate the relationship between initial H_2_O_2_ up-regulation and GA-induced PCD, the effects of different concentrations (100 μM or 1 mM) of H_2_O_2_ on GA-induced PCD were observed. We found that pretreatment with a low concentration (100 μM) of H_2_O_2_ was able to mimic the action of cobalt in the delay of GA-induced PCD (Figure 4). Both the GA and the ABA treatments led to a sensitization of wheat aleurone layers following 1 mM H_2_O_2_ pretreatment; and after 48 h of incubation, the proportions of dead cells were 97% and 10%, respectively (Figure 4B). In comparison, when treated with GA or ABA alone for 48 h, about 86% and 2% of the cells died, respectively.

**Figure 5:**
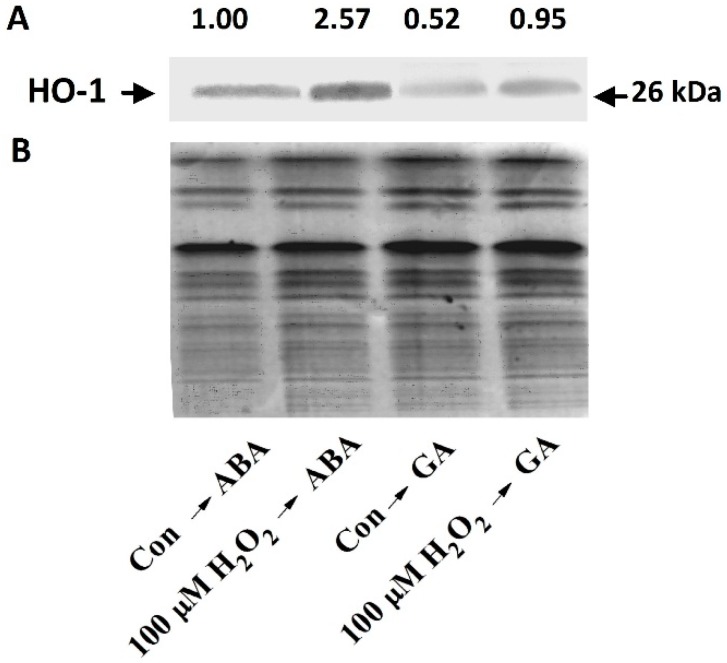
The effect of H_2_O_2_ pretreatment on HO-1 protein level. Wheat aleurone layers were pretreated with distilled water (Con) or 100 μM H_2_O_2_ for 6 h and then transferred to a medium containing 5 mM CaCl_2_ and 50 μM GA or ABA. HO-1 protein levels (**A**) were determined after 12 h of incubation. HO-1 protein levels were evaluated by western blotting; Coomassie Brilliant Blue-stained gels are presented to show that equal amounts of proteins extracts were loaded (**B**). The number above the band (**A**) illustrates the relative abundance of the corresponding HO-1 protein, compared with that of the ABA-treated sample.

A previous study showed that treatment with 1 mM H_2_O_2_ and GA or ABA caused a significant decrease in the expression of HO-1, HO-1 protein accumulation and HO activity [38]. Similar to this study, we observed that pretreatment with 1 mM H_2_O_2_ followed by treatment with GA or ABA for 12 h had inhibitory effects on *HO-1* gene expression (Figure 4B). In comparison, pretreatment with a lower concentration of H_2_O_2_ (100 μM) followed by treatment with GA or ABA caused marked increases in the levels of both *HO-1* transcripts and HO-1 protein (Figure 4B and Figure 5), which is similar to the responses observed with cobalt treatment (Figure 3A).

### 2.5. Decreased H_2_O_2_ Production Intensifies PCD in Wheat Aleurone Layers

**Figure 6:**
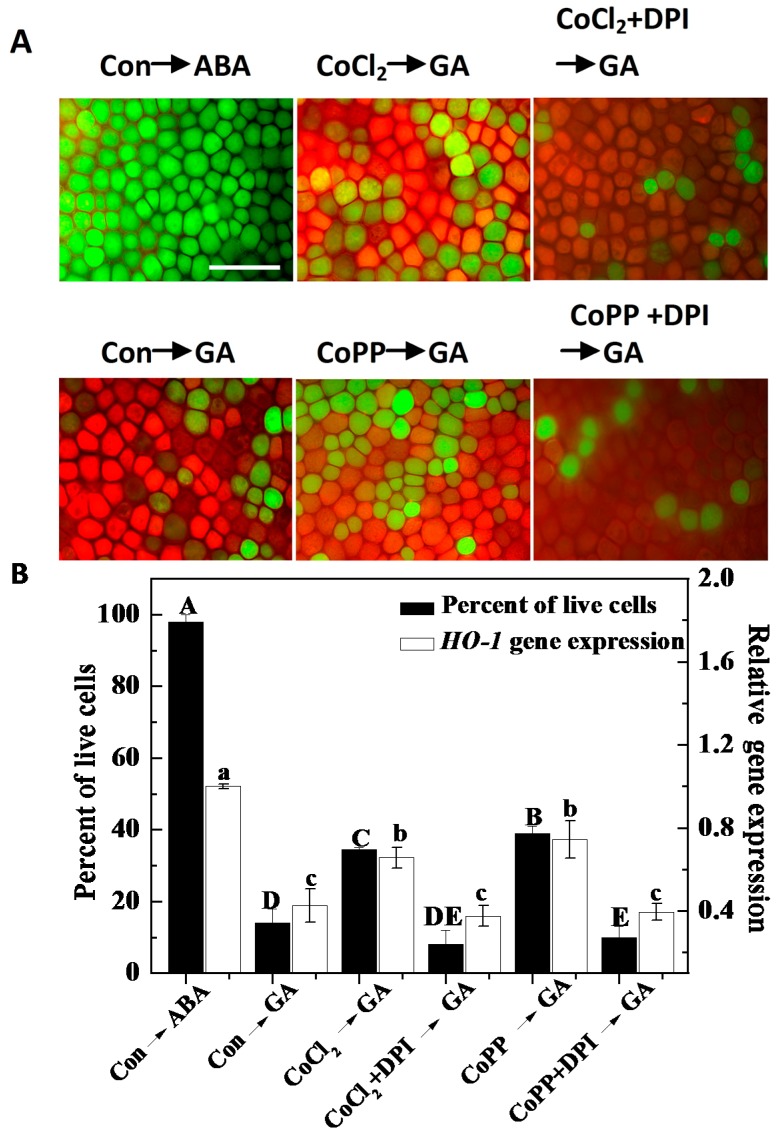
The effects of diphenyleneiodonium (DPI) (an inhibitor of NADPH oxidase) and cobalt on GA-induced PCD and *HO-1* gene expression. Wheat aleurone layers were pretreated with distilled water (Con), 50 μM CoCl_2_ or 50 μM CoPP in the presence or absence of 10 μM DPI, for 6 h, and then transferred to a medium containing 5 mM CaCl_2_ and 50 μM GA or ABA. (**A**) After 48 h of incubation, digital images of fluorescently-labeled wheat aleurone cells were taken; Live cells appear green and dead cells appear orange or red; Scale bar = 200 μm. The viability of cells following different treatments were quantified after 48 h of incubation from at least four aleurone layers (**B**, **left**). Wheat *HO-1* transcripts were measured after 12 h of treatment (**B**, **right**). Data are the means ± SD of at least four independent measurements from different experiments. Bars denoted by different letters are different significantly at *p* < 0.05 according to Duncan’s multiple test.

In order to further investigate the possibility of an interaction between the actions of H_2_O_2_ and cobalt, we assessed the effects of diphenyleneiodonium (DPI), a chemical inhibitor of NADPH oxidase, on the cobalt-induced alleviation of PCD. As shown in Figure 6, pretreatment with DPI resulted in an inhibitory effect on the action of cobalt in the delay of GA-induced PCD. For example, the percentage of live cells by pretreatment with CoCl_2_ or CoPPIX in the presence of DPI following the addition of GA for 48 h was reduced to 10% and 8%, respectively. These values are lower than those of the corresponding samples lacking DPI pretreatment. Subsequently, we noticed that the up-regulation of *HO-1* expression triggered by cobalt was notably decreased by DPI (Figure 6B), further confirming that the effects of cobalt on PCD and HO-1 expression are mediated specifically by H_2_O_2_.

### 2.6. Cobalt Action Is Sensitive to ZnPPIX

**Figure 7:**
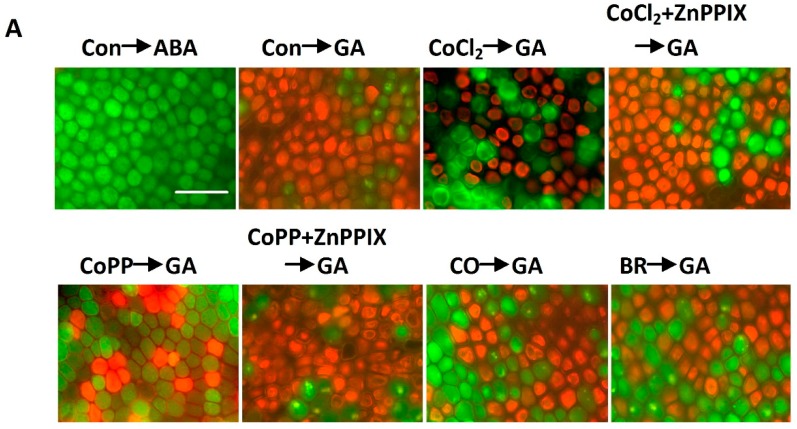

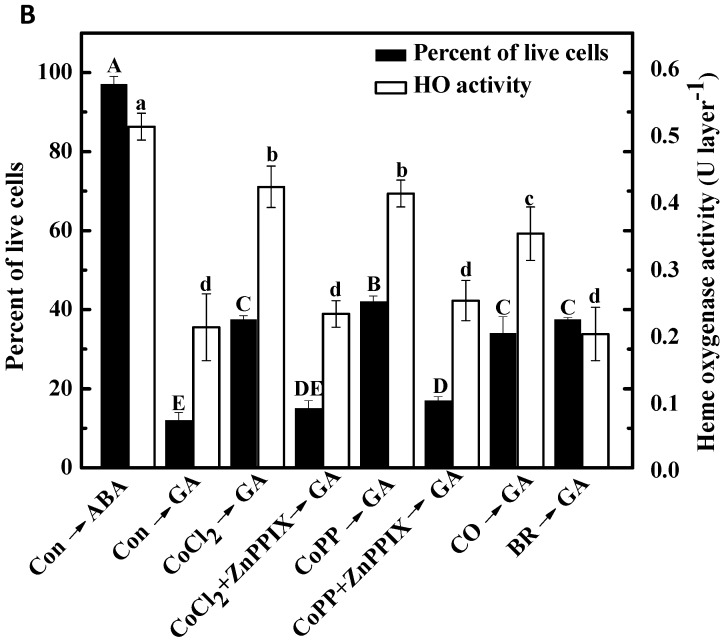
Decrease of HO activity related to the alleviation of PCD triggered by cobalt in wheat aleurone layers. Layers were pretreated with distilled water (Con), 50 μM CoCl_2_ or 50 μM CoPP, 1% CO aqueous solution, 10 μM bilirubin (BR), 100 μM ZnPPIX alone or a combination for 6 h and then transferred to a medium containing 5 mM CaCl_2_ and 50 μM GA or ABA. (**A**) Digital images of fluorescently-labeled wheat aleurone cells after 48 h of incubation were taken; Live cells appear green and dead cells appear orange or red; Scale bar = 200 μm; and (**B**, **left**) The viability of cells following different treatments were quantified for at least four aleurone layers; (**B**, **right**) HO activity was measured after 12 h of incubation. Data are the means ± SD of at least three independent measurements from different experiments. Within each set of experiments, bars denoted by different letters are significantly different at *p* < 0.05, according to Duncan’s multiple tests.

To confirm the possible role of HO-1 in the cobalt-induced delay of GA-induced PCD, we applied the potent HO-1 inhibitor, zinc protoporphyrin-IX (ZnPPIX), to GA-treated aleurone layers. As expected [38], HO activity was significantly inhibited by 100 μM ZnPPIX, in both ABA- and GA-treated layers (Figure 7B). Further experiments (Figure 7) showed that ZnPPIX not only prevented increases in HO activity, but also blocked the alleviation of PCD triggered by cobalt, indicating a requirement for HO-1 in the cobalt-induced delay of PCD in wheat aleurone layers.

The role of HO-1 in cobalt-induced responses was further examined by monitoring the phenotypes triggered by HO-1 end-products in wheat aleurone layers. CO, BR and Fe^2+^ are three end-products of HO-1 enzymatic function. Previous studies revealed that co-treatment with CO or BR delayed GA-induced PCD in wheat aleurone layers [38]. This conclusion was extended in the present work, showing that CO and BR pretreatments resulted in the alleviation of PCD induced by GA (Figure 7). These effects mimicked the cobalt response in PCD. Similar to the findings of a previous report [44], the increase in HO activity triggered by CO was approximately comparable to the cobalt effects, and no significant differences were observed between the BR-pretreated layers and the GA alone-treated layers (Figure 7B).

### 2.7. Decrease of H_2_O_2_ Accumulation Induced by Cobalt

**Figure 8:**
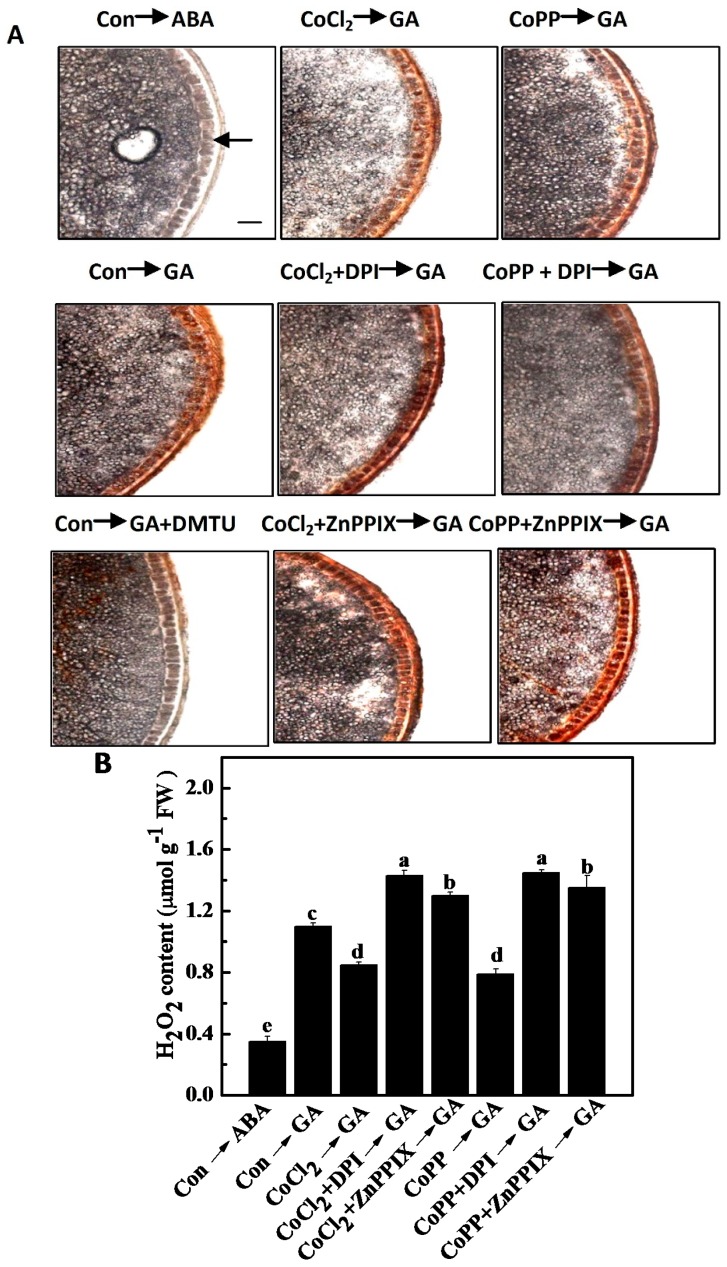
Production of H_2_O_2_ in wheat aleurone layers. Wheat aleurone layers were pretreated with distilled water (Con), 50 μM CoCl_2_ or 50 μM CoPP, 10 μM DPI, 100 μM ZnPPIX alone or a combination for 6 h and then transferred to a medium containing 5 mM CaCl_2_ and 50 μM ABA or GA. (**A**) After 24 h of incubation, samples were incubated with DAB (1 mg/mL) for 1 h. Arrows in the picture indicate aleurone layers. Scale bar = 50 μm; (**B**) H_2_O_2_ content was measured spectrophotometrically. Data are the means ± SD of at least three independent measurements from different experiments. Within each set of experiments, bars denoted by different letters are significantly different at *p* < 0.05, according to Duncan’s multiple tests.

To investigate the *in situ* accumulation and distribution of H_2_O_2_ in wheat aleurone layers, we examined the reaction of 3,3-diaminobenzidine (DAB) with H_2_O_2_ in aleurone layers; this reaction produces a brown polymerization product in the presence of peroxidase. Similar to previous reports, we observed that combined treatment with the H_2_O_2_ scavenger, *N*,*N*'-dimethylthiourea (DMTU), and GA significantly inhibited the accumulation of the brown polymerization product in wheat aleurone layers (Figure 8A). These results clearly implied that the brown signal that appeared following DAB staining was primarily caused by H_2_O_2_. When embryoless half-seeds were treated with GA for 24 h, H_2_O_2_ accumulation was more clearly visible in the aleurone layers than in the samples treated with ABA alone. The H_2_O_2_ accumulation was not observed in the endosperm [45].

A comparative slight accumulation of H_2_O_2_ was observed in cobalt-pretreated samples, suggesting a down-regulation of H_2_O_2_ production. By contrast, the reversal in the accumulation of H_2_O_2_ in GA-treated aleurone layers triggered by cobalt was blocked when DPI or ZnPPIX was added (Figure 8). Pretreatment with CO or BR almost completely blocked the accumulation of H_2_O_2_ induced by GA (Figure S4). Changes of H_2_O_2_ content under different treatments determined by spectrometric assays displayed similar tendencies. For example, the decrease of H_2_O_2_ production triggered by cobalt was notably blocked by the addition of DPI or ZnPPIX (Figure 8B). These results suggested the possibility that cobalt-induced HO-1 is critical in the regulation of H_2_O_2_ accumulation in wheat aleurone layers.

### 2.8. Changes of Antioxidant Enzyme Activities and Gene Expression

**Figure 9:**
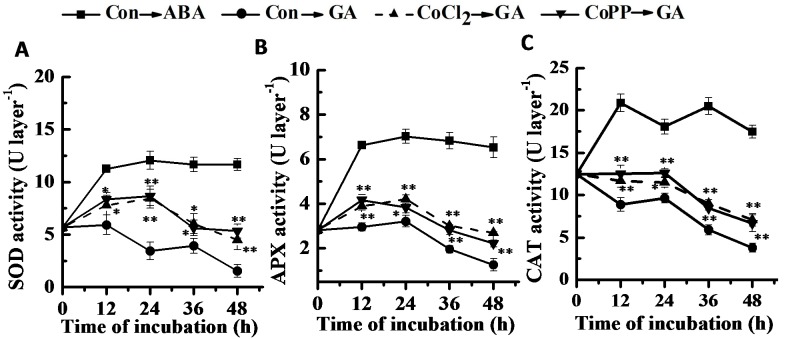
Cobalt prevents GA-induced decreases in SOD, ascorbate peroxidase (APX) and CAT activities in wheat aleurone layers. Wheat aleurone layers were pretreated with distilled water (Con), 50 μM CoCl_2_ or 50 μM CoPP for 6 h and then transferred to a medium containing 5 mM CaCl_2_ and 50 μM GA or ABA. SOD (**A**), APX (**B**) and CAT (**C**) activities were measured at the indicated time points. Data are the means ± SD of at least three independent measurements from different experiments. Within the same treatment time points, asterisks indicate significant difference (* *p* < 0.05 or ** p < 0.01) between CoCl_2_ or CoPP plus GA and GA alone-treated samples according to Student’s *t*-tests.

Previous results showed that GA-treated aleurone cells lose their ability to scavenge ROS and that this loss ultimately results in oxidative damage and PCD [5]. In our experiments, the activities of the antioxidant enzymes, superoxide dismutase (SOD), catalase (CAT) and ascorbate peroxidase (APX), and the transcript expression levels for the genes that encode these enzymes were measured. As shown in Figure 9, ABA treatment brought about an increased tendency of SOD, CAT and APX activities. By contrast, in comparison with GA-alone samples, pretreatment with CoCl_2_ or CoPP partially arrested the down-regulation of SOD, CAT and APX activities.

**Figure 10:**
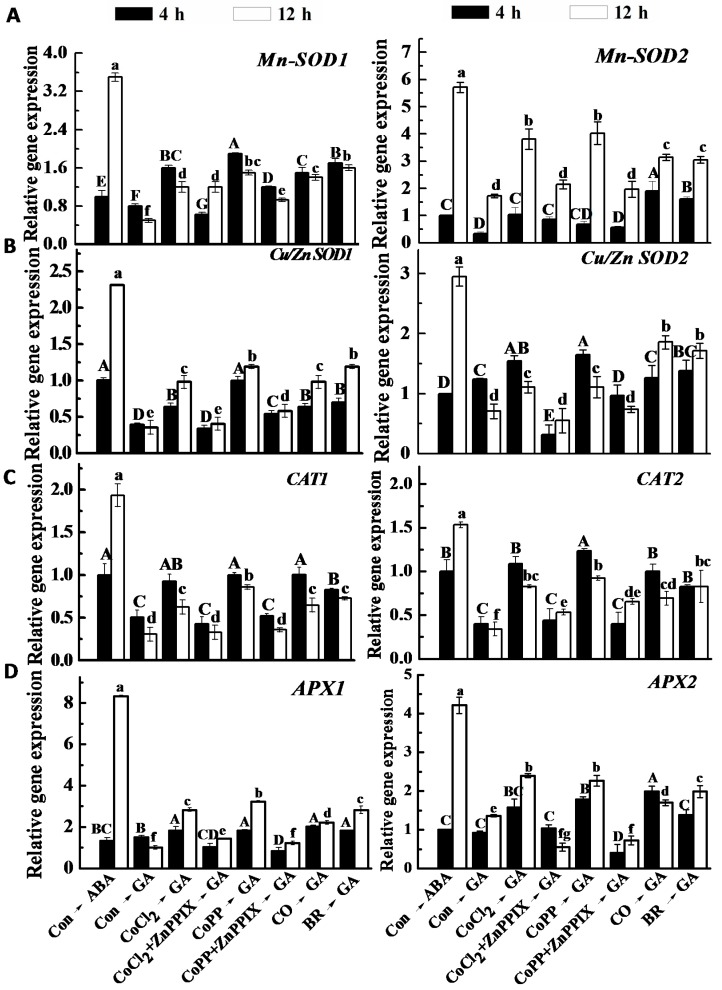
Expression patterns of antioxidant genes. Wheat aleurone layers were pretreated with distilled water (Con), 50 μM CoCl_2_, 50 μM CoPP, 1% CO aqueous solution, 10 μM bilirubin (BR), 100 μM ZnPPIX alone or a combination for 6 h and then transferred to a medium containing 5 mM CaCl_2_ and 50 μM GA or ABA, respectively. *Mn-SOD1/2* (**A**), *Cu/Zn SOD1/2* (**B**), *CAT1/2* (**C**) and *APX1/2* (**D**) transcripts were measured at the indicated time points. The expression levels of the genes are presented as values relative to ABA alone-treated samples (4 h). Data are the means ± SD of at least three independent measurements from different experiments. Within each set of experiment, bars denoted by different letters are significantly different at *p* < 0.05, according to Duncan’s multiple test.

qRT-PCR results further supported the observations detailed above. For example, pretreatment with cobalt partially increased *Cu/Zn SOD1/2*, *CAT1/2* and *APX1/2* gene expression, particularly at 12 h (Figure 10). This induction of gene expression was also observed in CO- and BR-pretreated layers. These results indicated that HO-1 was involved in the cobalt-induced up-regulation of the expression of antioxidant enzyme genes in the aleurone layers of wheat. All of the above changes were consistent with the measured H_2_O_2_ contents (Figure 8) and the alleviation of GA-induced PCD (Figure 1).

## 3. Discussion

Various cytotoxic and protective effects of cobalt have been reported for both animals and plants [9]. In this study, we discovered that low concentrations of cobalt (50 μM CoCl_2_ or CoPP) were able to delay PCD in wheat aleurone layers. We confirmed this finding by using FDA and FM 4-64 staining (Figure 1 and Figure S2) and by monitoring net O_2_ consumption (Figure 1C). This beneficial effect of cobalt in wheat aleurone layers was in agreement with previous studies that showed, for example, that cobalt can retard senescence in lettuce leaves in darkness [15], increase drought resistance in seeds of *Aesculus hippocastanum* [16] and regulate alkaloid accumulation in medicinal plants [17]. Cobalt is also known to cause hormone-induced phenotypes, such as the induction of adventitious rooting in tomato and cucumber cuttings and lateral root development in rice and tomato seedlings [12,13,14]. Normally, hormonal-like substances usually exhibited dose-dependent effects. We found that a higher concentration (500 μM) of cobalt was able to accelerate GA-induced PCD. This might be ascribed to the possibility that high concentrations of cobalt hamper RNA synthesis and interfere with heme biosynthesis, which has been shown previously in plants and in fungi [9,46]. Combined with no significant or negative response following treatment with a lower (5 μM) or higher (500 μM) concentration of cobalt in the delay of GA-induced PCD, our results suggested that the positive role of cobalt in the regulation of PCD was effective in a narrow range of concentrations. This finding is similar to what is known about the action of hemin, an inducer of HO-1 expression [38].

Cobalt’s effect on cell viability can be mimicked by low doses of H_2_O_2_ (Figure 4), CO or BR (Figure 7). CO and BR are two catalytic end-products of HO-1, an antioxidant enzyme that has been characterized recently in animals and plants [19,38,43,47,48]. Previous studies have shown that cobalt can induce HO activity and/or its gene expression in animals [49,50,51]. The up-regulation of HO expression was found to be involved in modulating oxidative status [52]. Consistent with the relationships delineated in these studies, we found that in the aleurone layers of wheat, the initial induction of H_2_O_2_ production that occurs in the cobalt-induced delay of PCD subsequently attenuates the ultimate level of H_2_O_2_ accumulation via the increased antioxidative enzymatic capacity that results from increased HO-1 expression.

Cobalt may act in an HO-1-dependent fashion. There is an obvious relationship between the level of *HO-1* transcripts and the degree of severity of PCD: As HO-1 levels increase, the severity of PCD decreases (*i.e*., fewer cells die). Time course analysis showed that GA treatment obviously slowed down the increase in *HO-1* gene expression, as compared to the increase seen in ABA-treated aleurone samples (Figure 3A). Pretreatment with ZnPPIX, a potent inhibitor of HO, was able to reverse the up-regulation of HO activity, as well as alleviate the GA-induced PCD triggered by cobalt (Figure 7). We also noticed that the cell death in ABA-treated aleurone layers was less susceptible to ZnPPIX than the cell death of the GA-treated layers, although there were significant decreases in HO activity (Figure 7B). Similarly, ABA-treated wheat and barley aleurone layers are known to be less susceptible to H_2_O_2_ than the GA-treated cells [38,53]. Combined with previous results showing that cobalt-induced lateral root formation in tomato and rice seedlings occurs in a HO-1-dependent manner [13,14], our results point to a causal relationship between cobalt and HO-1 in the delay of PCD.

It is well known that ROS are key players in many types of PCD. Previous studies have shown that increases in intracellular ROS accumulation caused by the rapid reduction of *CAT*, *APX* and *SOD* gene expression and activities in barley aleurone layers mediate GA-induced PCD [5,6,53]. This conclusion is supported by a recent finding that, in wheat aleurone layers, HO-1 delays PCD by modulating H_2_O_2_ metabolism [38]. As expected, when using DMTU as a scavenger of H_2_O_2_ (Figure 8), we found that endogenous ROS, particularly H_2_O_2_, are inducers of PCD in the aleurone layers of cereal seeds. Similar to previous reports in barley [6,53], we showed that PCD in wheat aleurone layers is mediated by intracellular H_2_O_2_ accumulation at 24 h of GA treatment. Lower antioxidant capacity in wheat aleurone layers, including the transcriptional and enzymatic levels of representative antioxidant enzymes, mediate GA-induced PCD (Figure 9 and Figure 10). The alleviation of PCD caused by cobalt can be directly attributed to the decrease of H_2_O_2_ accumulation in the tissue (Figure 8). This deduction is supported by the finding that the induced gene expression of antioxidant enzymes triggered by cobalt, including *Mn-SOD1/2*, *Cu/Zn SOD1*, *CAT1/2* and *APX1/2* (Figure 10), are consistent with decreased H_2_O_2_ accumulation and the alleviation of PCD. Contrasting phenomena were mostly observed when ZnPPIX was used as a co-treatment. Therefore, our results are consistent with the observation reported by Jabs that showed that the cell death that occurs during the pathogen-induced hypersensitive response is accompanied by an increase in the production of ROS and that this increase is mainly due to the activation of a plasma membrane-associated NAD(P)H oxidase, which mediates H_2_O_2_ production [54]. Given that the decreased in intracellular ROS levels modulated by antioxidant enzymes may be an important component in the alleviation of PCD in barley aleurone layers [5], we speculate that the decrease of H_2_O_2_ production achieved by the enhancement of antioxidant enzymes may act downstream of cobalt in the alleviation of PCD in wheat aleurone layers.

In animals, several lines of evidence point to the fact that H_2_O_2_ and HO-1 not only modulate each other’s functions within the cell, but also depend on each other to impart their effects in most conditions [43]. A previous study showed that when GA-treated (24 h) barley aleurone cells were incubated with 325 mM H_2_O_2_, virtually all of the cells died within 60 min [53]. In an earlier study, we noticed that the application of 1 mM H_2_O_2_ not only decreased *HO-1* gene expression, but also accelerated PCD in wheat aleurone layers [38]. In the present study, however, 1 mM H_2_O_2_ pretreatment for 6 h was found to accelerate GA-induced PCD in wheat aleurone layers, and 100 µM H_2_O_2_ was found to delay GA-induced PCD (Figure 4). These results confirmed the supposition that H_2_O_2_ might be a double-edged sword that is at least partially dependent on its concentration or the treatment periods of exogenously applied H_2_O_2_; and the effects of H_2_O_2_ may even differ among plant species. This cytoprotective role of H_2_O_2_ at low doses might be related to the up-regulation of *HO-1* expression (Figure 5). Similarly, ROS-triggered up-regulation of *HO-1* gene expression was observed in soybean [35], wheat [36] and rice plants [55]. Thus, the results mentioned above further indicate that the maintenance of a suitable level of intracellular H_2_O_2_ during cobalt pretreatment (Figure 6) is vital for the alleviation of PCD.

Based on our results, we suggest that this beneficial role in delaying GA-induced PCD may result from cobalt’s ability to up-regulate *HO-1* gene expression and down-regulate intracellular H_2_O_2_ accumulation. In particular, our findings highlight that HO-1 may serve as an important component in the complex signaling networks involved in plant responses to cobalt exposure [13,14,49,50]. Our work and the ongoing analyses of the roles of other cobalt target genes/components in the cobalt-triggered alleviation of GA-induced PCD should open new windows to our understanding of how cobalt ultimately functions in plants.

## 4. Experimental Section

### 4.1. Plant Materials and Chemicals

Aleurone layers were prepared from de-embryonated wheat (*Triticum aestivum* “Yangmai 13”) grains, as previously described [38,56,57]. Briefly, de-embryonated half-grains were surface-sterilized in 1% sodium hypochlorite solution for 20 min and then rinsed extensively with sterile water at least three times. Fifty de-embryonated half-grains per petri dish were then imbibed in sterile water at 25 ± 1 °C for 48 h. Aleurone layers were isolated from the imbibed grains by gently removing the starchy endosperm under sterile conditions; these non-starchy aleurone layers were used in the various treatments.

All chemicals were purchased from Sigma (St Louis, MO, USA), unless stated otherwise. Cobalt chloride (CoCl_2_) and cobalt protoporphyrin IX (CoPP) were used at the indicated concentrations [13,58,59]. The other chemicals used for treatments were zinc protoporphyrin-IX (ZnPPIX, 50 µM), BR (10 µM) [38,60], diphenyleneiodonium (DPI, 10 µM) [30] and *N*,*N*'-dimethylthiourea (DMTU, Fluka, 5 mM) [38]. The fluorescent probes, fluorescein diacetate (FDA) and *N*-(3-triethylammoniumpropyl)-4-(6-[4-(diethylamino) phenyl]-hexatrienyl) pyridinium dibromide (FM 4-64), were purchased from ICN Biomedicals Inc. and Life Inc. (Molecular Probes), respectively [5,37]. The concentrations used with the aforementioned chemicals were chosen based on pilot experiments in which the concentrations required to induce significant responses were determined.

### 4.2. Preparation of 1% CO Aqueous Solution

The preparation of the CO aqueous solution with 1% saturation was carried out according to the methods described in our previous work [38,60].

### 4.3. Determination of Cell Viability

The viability of wheat aleurone layer cells was determined by double staining with fluorescein diacetate (FDA, 2 μg mL^−1^ in 5 mM CaCl_2_) for 15 min and then staining with FM 4-64 (20 μM in 5 mM CaCl_2_) for 3 min [5,37,38]. Layers were observed with a flfluorescent microscope (Axio Imager A1; Carl Zeiss, Oberkochen, Germany) using a 20× objective lens. Images of the fluorescent signal were captured using a digital camera. Four or five fields from one aleurone layer were randomly selected, and at least four different aleurone layers were evaluated for each of the treatments. Live and dead cells were counted to determine the proportion of viable cells.

### 4.4. O_2_ Consumption

Ten aleurone layers for per treatment were used to measure O_2_ consumption using an O_2_-sensitive electrode (oxy-lab, Hansatech, Norfolk, UK) [37,38]. Briefly, the aleurone layers of the various treatments were washed three times in sterile water and then transferred to a measuring chamber containing 3 mL of sterile distilled water at least 20 min prior to determining the rate of O_2_ consumption.

### 4.5. Determination of HO Activity and Western Blot Analysis for the HO-1 Protein

Aleurone layers (30) were used to determine wheat HO activity and HO-1 protein levels by using previously described methods [21,38,61,62]. One unit of activity (U) was calculated by taking the quantity of the enzyme to produce 1 nmol BV per 30 min. The antibody used was rabbit polyclonal antibody raised against recombinant mature wheat HO-1 with a molecular mass of 26 kDa [38]. Fifty micrograms of protein from homogenate extracts were subjected to SDS-PAGE using a 12.5% acrylamide resolving gel [61]. Separated proteins were then transferred to polyvinylidene difluoride (PVDF) membranes, and non-specific binding of antibodies was blocked with 5% non-fat dried milk in phosphate-buffered saline (PBS, pH 7.4) for 2 h at room temperature. Membranes were then incubated overnight at 4 °C with primary antibodies diluted 1:200 in PBS buffer plus 1% non-fat dried milk. Immune complexes were detected using horseradish peroxidase (HRP)-conjugated goat anti-rabbit immunoglobulin G. The color was developed with a solution containing 3,3-diaminobenzidine tetrahydrochloride (DAB) as the HRP substrate. For quantification of HO-1 protein levels, filters were scanned (Uniscan B700^+^, Tsinghua Unigroup Ltd., Beijing, China) and band intensities were determined with Quantity One v4.4.0 software (Bio-Rad, Hercules, CA, USA).

### 4.6. Determination of the Activities of Other Enzymes

Aleurone layers (10) were used to determine α-amylase activity in the incubation medium using the starch-iodine procedure [37,38,63,64]. One unit of α-amylase activity (U) is defined as a change of one absorbance unit at 620 nm min^−1^. Furthermore, aleurone layers (15) were used to determine the activities of superoxide dismutase (SOD), ascorbate peroxidase (APX) and catalase (CAT) according to the methods described in our previous reports [22,38,65]. Total superoxide dismutase (SOD) activity was measured on the basis of its ability to reduce nitroblue tetrazolium (NBT) by superoxide anions generated by the riboflavin system under illumination. One unit of SOD (U) was defined as the amount of crude enzyme extract required to inhibit the reduction rate of NBT by 50%. CAT activity was spectrophotometrically measured by monitoring the consumption of H_2_O_2_ (ε = 39.4 mM^−1^ cm^−1^) at 240 nm. APX activity was determined by monitoring the decrease at 290 nm (ε = 2.8 mM^−1^ cm^−1^). One unit (U) of CAT or APX was defined as the decomposition of 1 µmol H_2_O_2_ or AsA min^−1^ [38].

### 4.7. RNA Isolation, cDNA Synthesis and qRT-PCR Analysis

Total RNA was isolated using a method described in our previous work [66]. Following isolation, the RNA samples were treated with RNAase-free DNase (TaKaRa Bio Inc., Dalian, China) to eliminate traces of DNA, followed by quantification using a NanoDrop 2000 spectrophotometer (Thermo Fisher Scientific, Wilmington, DE, USA). cDNA was synthesized from 2 μg aliquots of RNA using oligo(dT) primer and avian myeloblastosis virus (AMV) reverse transcriptase XL (TaKaRa, Dalian, China). The transcript expression levels of each gene were measured via qRT-PCR using a Mastercycler^®^ ep *realplex* real-time PCR system (Eppendorf, Hamburg, Germany) according to the manufacturer’s instructions, using SYBR^®^ *Premix* *Ex* *Taq*™ (TaKaRa,) and the primers detailed in Table S1. The relative expression levels of each gene in the aleurone layers were determined relative to the corresponding control samples at the indicated time points or conditions, after normalization to the transcript levels of actin [61].

### 4.8. Visualization and Measurement of H_2_O_2_ Production

Visualization of H_2_O_2_ in embryoless half-seeds was performed as described previously [45]. After treatment, the embryoless half-seeds were sectioned horizontally into 60 µm sections with a rapid freezing microtome (Leica CM1950) and then stained with 3,3-diaminobenzidine (DAB; 1 mg/mL) for 1 h. After staining, images were observed with a microscope (Axio Imager A1; Carl Zeiss, Germany) using a ×20 objective lens.

H_2_O_2_ content in aleurone layers (30) was spectrophotometrically analyzed according to previously described methods [38,67,68]. The method was based on the peroxide-mediated oxidation of Fe^2+^, followed by the reaction of Fe^3+^ with xylenol orange (Sigma). Thirty aleurone layers were used to determine H_2_O_2_ content for each treatment. Standard curves were obtained by adding variable amounts of H_2_O_2_.

### 4.9. Statistical Analysis

Values are shown as the means ± SD of at least three independent experiments for each treatment. For statistical analysis, either Student’s *t*-tests (*p* < 0.05 or *p* < 0.01) or Duncan’s multiple test (*p* < 0.05) were selected where appropriate.

## 5. Conclusions

Cobalt exhibited a cytoprotective role in the alleviation of GA-induced PCD in wheat aleurone layers. The initial H_2_O_2_ production induced by cobalt operates through an HO-1-mediated antioxidant system, ultimately attenuating the level of H_2_O_2_ accumulation and GA-induced PCD.

## Supplementary Materials

Supplementary Table and Figures can be found at http://www.mdpi.com/1422-0067/15/11/21155/s1.
